# Supplementary material for: A hPSC-based platform to discover gene-environment interactions that impact human β-cell and dopamine neuron survival
Source: Nat Commun. 2018 Nov 16;9:4815. doi: 10.1038/s41467-018-07201-1 (PMC6240096; doi:10.1038/s41467-018-07201-1)
Supplement: Supplementary file 1 — Supplementary Information [file 41467_2018_7201_MOESM1_ESM.pdf]

Supplementary Figure 1

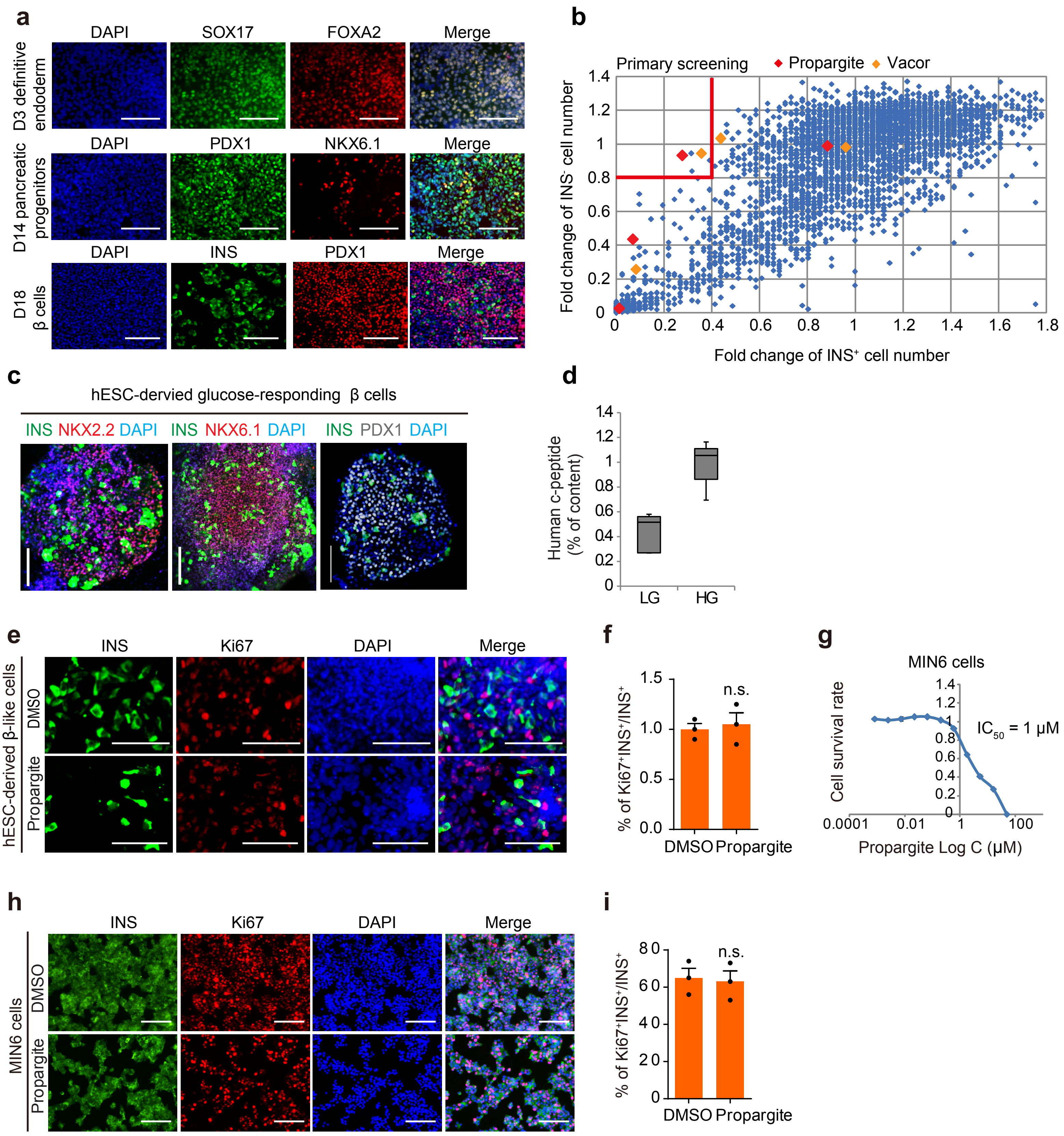

Supplementary information.

Supplementary Figure 1. Primary screen and validation of hit compounds.
(**a**) Immunocytochemistry analysis of hESC-derived definitive endoderm, pancreatic progenitors, endocrine cells and INS^+ cells using the β-cell differentiation protocol 1.
(**b**) Results of ToxCast library screening using the β-cell differentiation protocol 1. The red or orange squares highlight the positive hit compounds. (**c, d**) Confocal imaging analysis (c) and glucose stimulated insulin section (d) to characterize hESC-derived glucose-responding β-like cells generated using β-cell differentiation protocol 2. Scale bar = 150 µm. Values presented as the box plot. Centre line, the bounds of box and whiskers present the median, the first, third quartile, minimal and maximum, repsectively. (**e, f**) Representative images (e) and cell proliferation rate (f) of DMSO or 1.6 µM propargite treated β-like cells (n=3). Pancreatic β-like cells were stained for INS (green), proliferating cells were stained for Ki67 (red) and all the cells were counterstained by DAPI (blue). Scale bars, 200 µm. Cell proliferation rates were defined as the percentage of $Ki67^+INS^+$ cells/INS^+ cells. Values presented as mean±S.D. (**g**) IC_{50} of propargite on MIN6 cells. (**h, i**) Representative images (h) and cell proliferation rates (i) of DMSO or 1.6 µM propargite treated MIN6 cells. MIN6 cells were stained for INS (green), proliferating cells were stained for Ki67 (red) and all the cells were counterstained by DAPI (blue). Scale bars, 200 µm. Values presented as mean±S.D. n.s. indicates a non-significant difference.

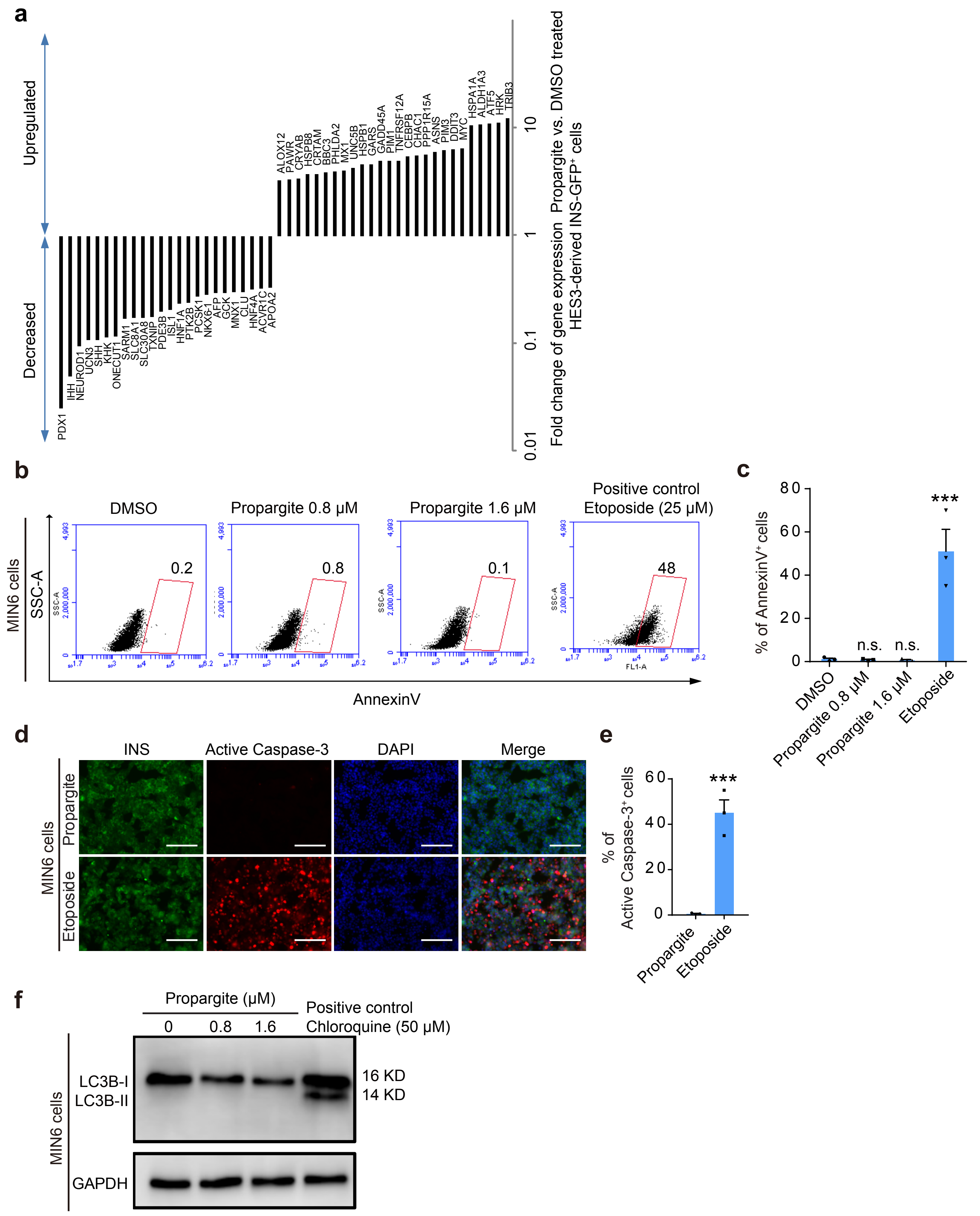
a
Upregulated
Decreased
Fold change of gene expression Propargite vs. DMSO treated HES3-derived INS-GFP+ cells
10
1
0.1
0.01
PDX1
IHH
NEUROD1
UCN3
SHH
KHK
ONECUT1
SARM1
SLC8A1
SLC30A8
TXNIP
PDE3B
ISL1
HNF1A
PTK2B
PCSK1
NKX6-1
AFP
GCK
MNX1
CLU
HNF4A
ACVR1C
APOA2
ALOX12
PAWR
CRYAB
HSPB8
CRTAM
BBC3
PHLDA2
MX1
UNC5B
HSPB1
GARS
GADD45A
PIM1
TNFRSF12A
CEBPB
CHAC1
PPP1R15A
ASNS
PIM3
DDIT3
MYC
HSPA1A
ALDH1A3
ATF5
HRK
TRIB3
b
DMSO
Propargite 0.8 μM
Propargite 1.6 μM
Positive control
Etoposide (25 μM)
MIN6 cells
SSC-A
0.2
0.8
0.1
48
AnnexinV
c
% of AnnexinV+ cells
80
60
40
20
0
***
n.s.
n.s.
DMSO
Propargite 0.8 μM
Propargite 1.6 μM
Etoposide
d
INS
Active Caspase-3
DAPI
Merge
MIN6 cells
Propargite
Etoposide
e
% of Active Caspase-3+ cells
60
40
20
0
***
Propargite
Etoposide
f
Propargite (μM)
0
0.8
1.6
Positive control
Chloroquine (50 μM)
MIN6 cells
LC3B-I
LC3B-II
16 KD
14 KD
GAPDH

Supplementary Figure 2. Propargite-induced β-cell death is not due to cell apoptosis or autophagy.

(**a**) Up/down-regulated genes (fold change >3) in the 1.6 μM propargite-treated *INS*-GFP^+ cells. (**b, c**) Flow cytometry analysis (b) and quantification (c) of the percentage of $AnnexinV^+$ cells for different doses of propargite-treated MIN6 cells (n=3). 25 μM Etoposide was used as a positive control. Values presented as mean±S.D. (**d, e**) Immunocytochemistry analysis (d) and quantification (e) of the percentage of Active Caspase-3^+ cells in 1.6 μM propargite treated MIN6 cells (n=3). 25 μM Etoposide used as a positive control. Scale bars, 200 μm. Values presented as mean±S.D. (**f**) Western blot analysis of autophagy marker (LC3B-II) after 0.8 μM or 1.6 μM propargite exposure in MIN6 cells. 50 μM Chloroquine was used as a positive control. GAPDH was used as a loading control. n.s. indicates a non-significant difference. *p* values calculated by unpaired two-tailed Student's t-test was ***$p<0.001$.

Supplementary Figure 3

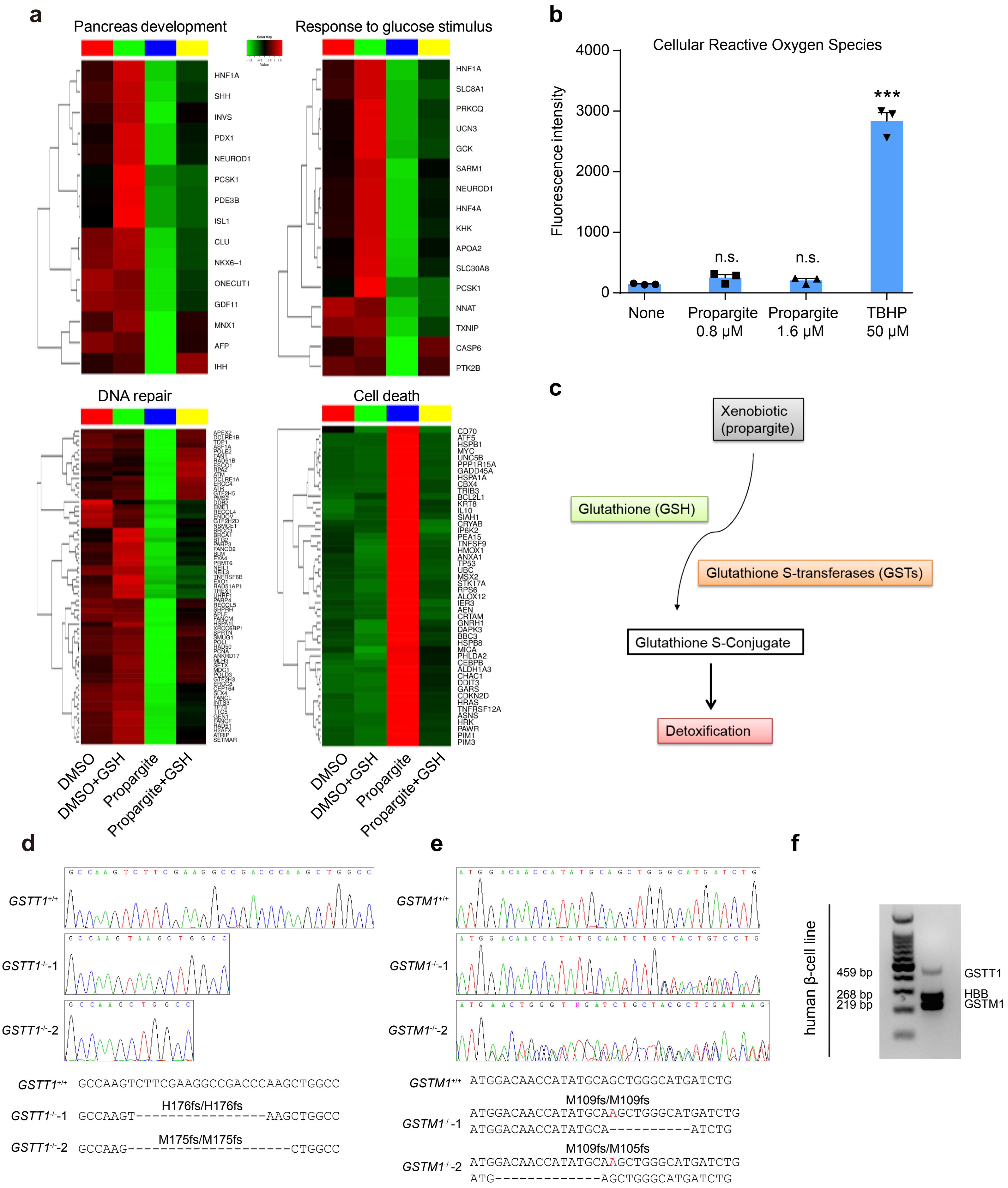

Supplementary Figure 3. GSH rescues the transcriptional alteration of propargite treated INS^{+} cells, through an anti-oxidant independent manner.

(**a**) Transcript profiling showed that the transcriptional alteration in propargite-treated *INS*-GFP^{+} cells were largely reversed by GSH, including gene expression related to pancreas development, pancreas function, DNA repair, and cell death. (**b**) Cellular Reactive Oxygen Species detection for different doses of propargite-treated MIN6 cells (n=3). 50 μM TBHP was used as a positive control. (**c**) Suggested mechanism for the GSH-dependent propargite detoxification pathway in rats. (**d, e**) Sequences of two representative biallelic mutants (*GSTT1*$^{-/-}$-1 and *GSTT1*$^{-/-}$-2) with frameshift mutations on *GSTT1* (d) and two representative biallelic mutants (*GSTM1*$^{-/-}$-1 and *GSTM1*$^{-/-}$-2) with frameshift mutations on *GSTM1* (e). (**f**) Genotype analysis of *GSTM1* and *GSTT1* in EndoC-βH1 cells. Values presented as mean±S.D. n.s. indicates a non-significant difference. *p* values calculated by unpaired two-tailed Student's t-test was ***$p<0.001$.

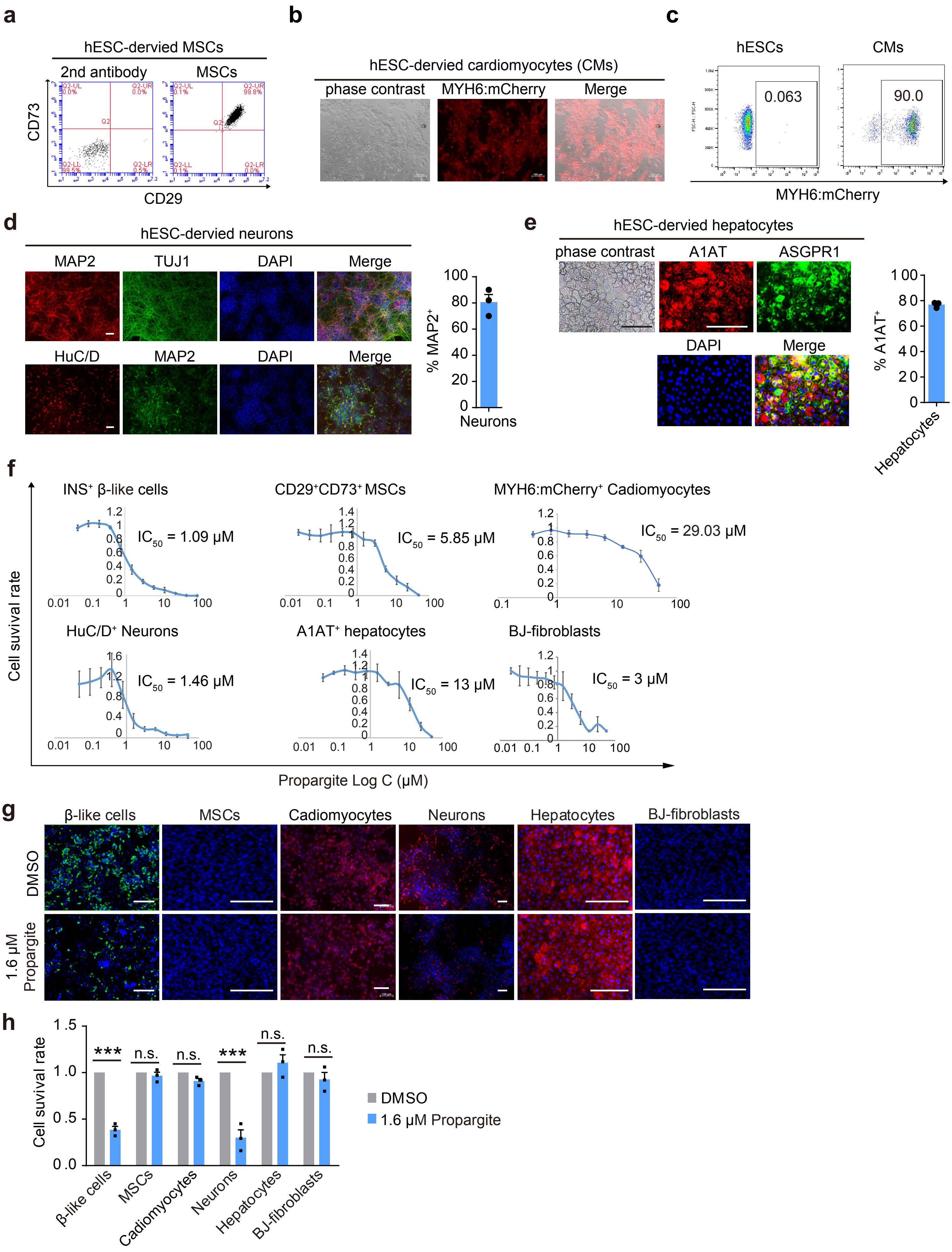
a
hESC-dervied MSCs
2nd antibody
MSCs
CD73
CD29
b
hESC-dervied cardiomyocytes (CMs)
phase contrast
MYH6:mCherry
Merge
c
hESCs
CMs
0.063
90.0
MYH6:mCherry
d
hESC-dervied neurons
MAP2
TUJ1
DAPI
Merge
HuC/D
MAP2
DAPI
Merge
% MAP2+
Neurons
e
hESC-dervied hepatocytes
phase contrast
A1AT
ASGPR1
DAPI
Merge
% A1AT+
Hepatocytes
f
INS+ β-like cells
IC50 = 1.09 μM
CD29+CD73+ MSCs
IC50 = 5.85 μM
MYH6:mCherry+ Cadiomyocytes
IC50 = 29.03 μM
HuC/D+ Neurons
IC50 = 1.46 μM
A1AT+ hepatocytes
IC50 = 13 μM
BJ-fibroblasts
IC50 = 3 μM
Cell suvival rate
Propargite Log C (μM)
g
β-like cells
MSCs
Cadiomyocytes
Neurons
Hepatocytes
BJ-fibroblasts
DMSO
1.6 μM Propargite
h
Cell suvival rate
***
n.s.
n.s.
***
n.s.
n.s.
DMSO
1.6 μM Propargite
β-like cells
MSCs
Cadiomyocytes
Neurons
Hepatocytes
BJ-fibroblasts

Supplementary Figure 4. Assessment of the toxic effect of propargite on different type of cells derived from hESCs, showing that HuC/D^{+} neurons are highly susceptible to propargite.

(**a**) Flow cytometry characterization of CD29^{+} and CD73^{+} mesenchymal stem cells (MSCs) derived from H1 hESCs. (**b-c**) microscope image (b) and flow cytometry (c) characterization of cardiomyocytes expressing MYH6+ derived from a MYH6:mCherry reporter hESC line. (**d-e**) Immunocytochemistry characterization of MAP2^{+}/TUJ1^{+} and HuC/D^{+}/MAP2^{+} neurons (d) and A1AT^{+}/ASGPR1^{+} hepatocytes (e) derived from H1 hESCs. All the cells were counterstained by DAPI (blue). Scale bars, 100 μm. (**f**) Inhibition curve of propargite on pancreatic β-like cells, MSCs, cardiomyocytes, neurons, and hepatocytes derived from hESCs, and BJ-fibroblasts (n=3). Values presented as mean±S.D. (**g, h**) Representative images (g), and survival rate (h) of different types of cells derived from hESCs and BJ-fibroblasts in response to 1.6 μM propargite (n=3). All the cells were counterstained by DAPI (blue). Scale bars, 100 μm. Values presented as mean±S.D. n.s. indicates a non-significant difference. *p* values calculated by unpaired two-tailed Student's t-test was ***$p<0.001$.

Supplementary Figure 5

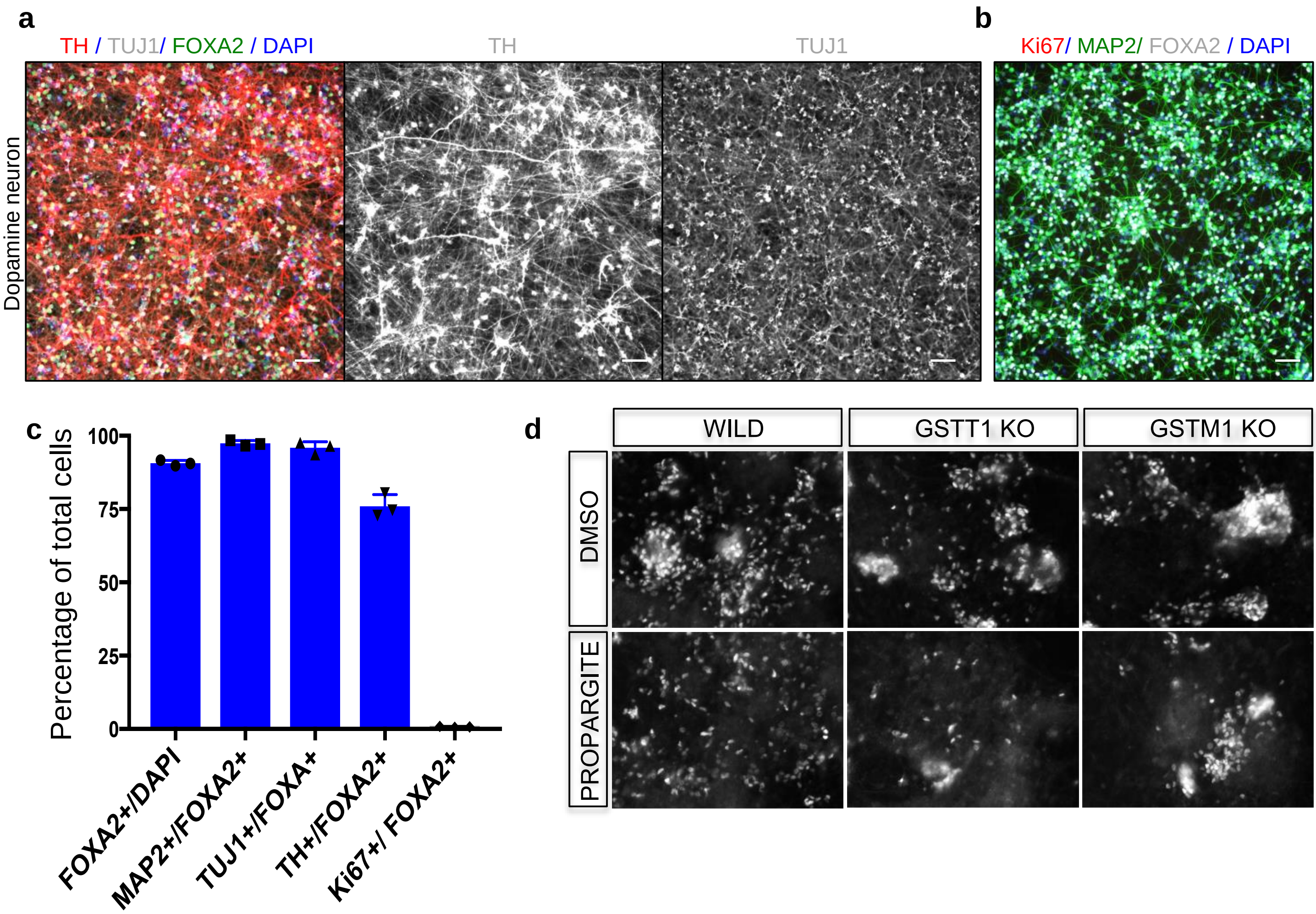

Supplementary Figure 5. Characterization of hESC-derived midbrain dopamine (mDA) neurons treated with/without propargite.

(**a-c**) More than 90% of hESC-derived mDA differentiated cells are FOXA2 positive. Among FOXA2 positive cells, more than 95% are MAP2 (a pan-neuronal marker) positive and 75% are TH (a mature DA marker which encodes late limit enzyme for dopamine production) positive, but less than 1% are Ki-67 (a marker for cell proliferation) positive. Values presented as mean±S.D. (**d**) Representative images of mDA differentiated $FOXA2^{+}$ cells treated with DMSO or propargite (3 μM) derived from isogenic wild type, *GSTT1*$^{-/-}$, and *GSTM1*$^{-/-}$ H1 hESC.

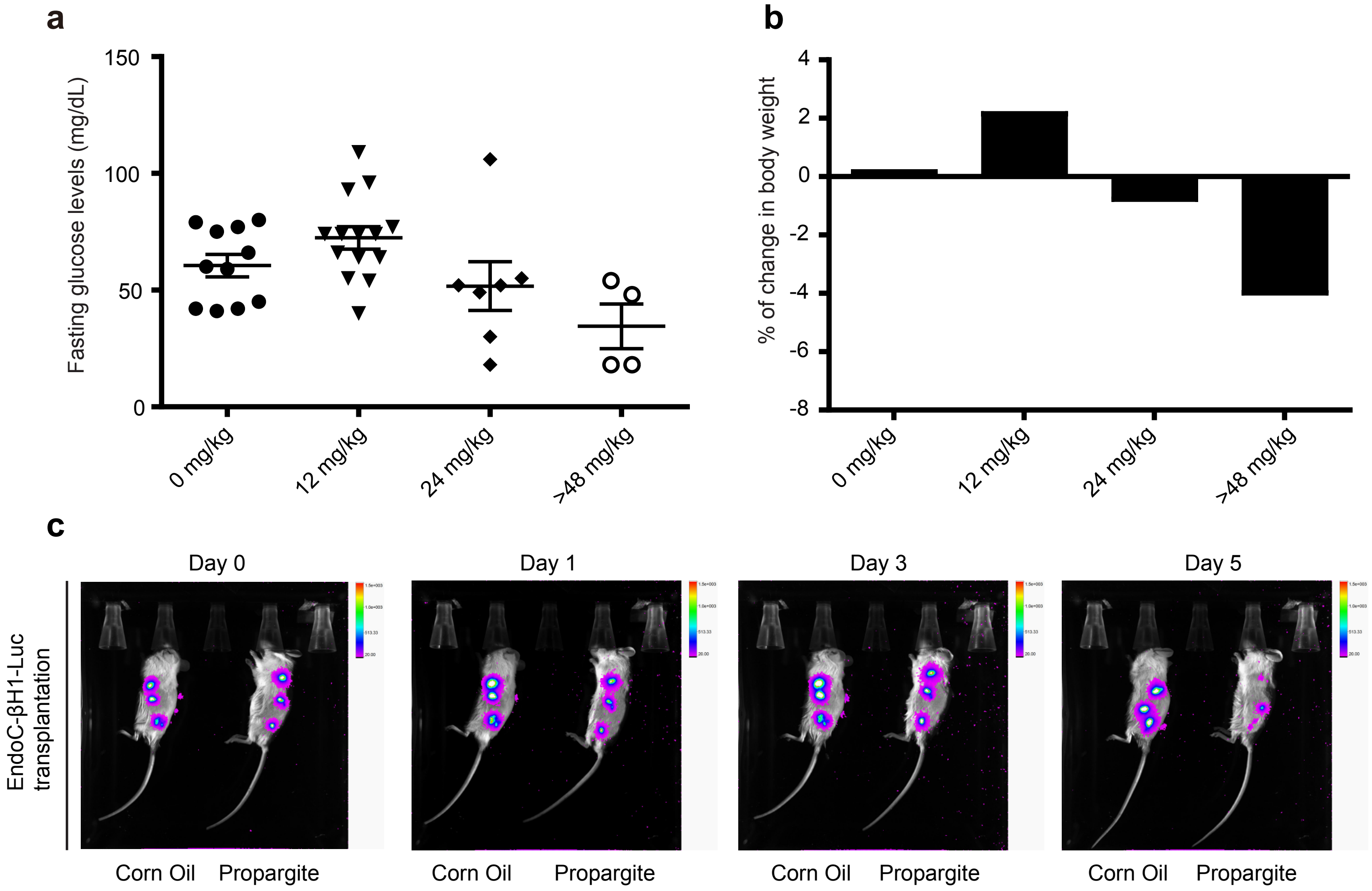
a
Fasting glucose levels (mg/dL)
150
100
50
0
0 mg/kg
12 mg/kg
24 mg/kg
>48 mg/kg
b
% of change in body weight
4
2
0
-2
-4
-6
-8
0 mg/kg
12 mg/kg
24 mg/kg
>48 mg/kg
c
Day 0
Day 1
Day 3
Day 5
EndoC-βH1-Luc transplantation
Corn Oil
Propargite
Corn Oil
Propargite
Corn Oil
Propargite
Corn Oil
Propargite

Supplementary Figure 6. Propargite induces β-cell toxicity *in vivo*.

(**a**, **b**) Fasting mouse blood glucose level levels (a) and % of change in body weight (b) in corn oil (control) or different doses of propargite treated mice. Values presented as mean±S.D. (c) Representative images of *in vivo* imaging for luciferase signals from EndoC-βH1-Luc cells transplanted mice administrated with corn oil (vehicle) or propargite during the 5-day course.

Fig.2c

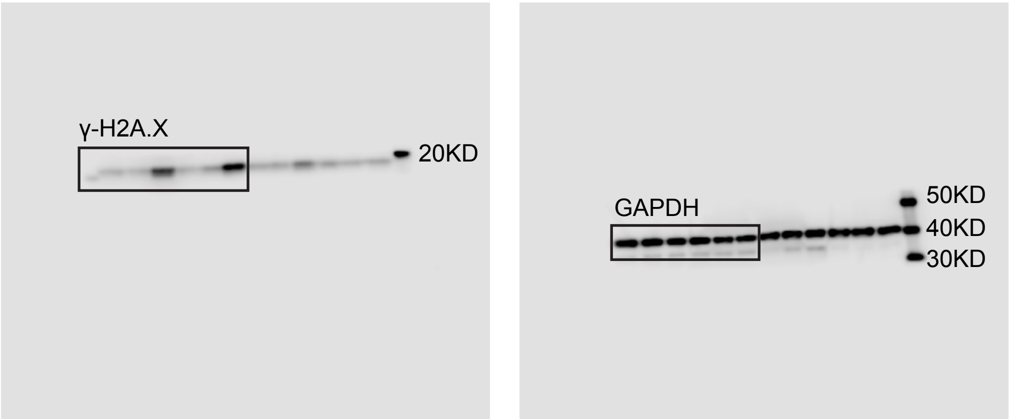

Fig.2d

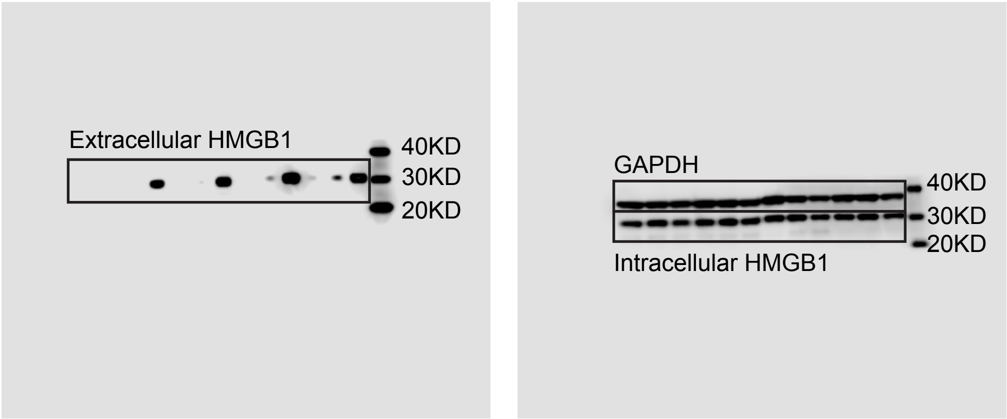

Fig.2e

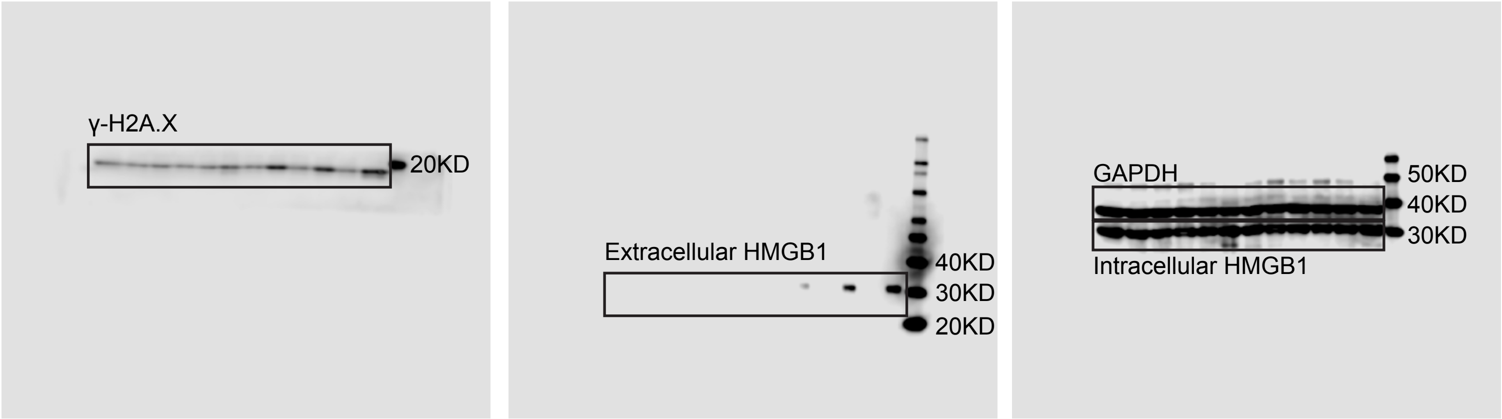

Fig.2m

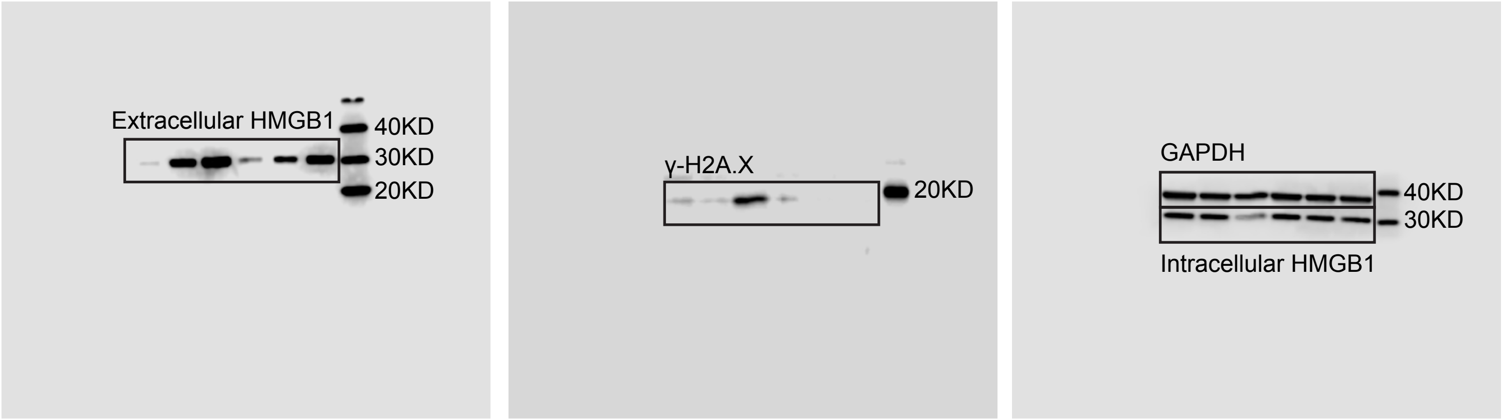

Fig. 3d

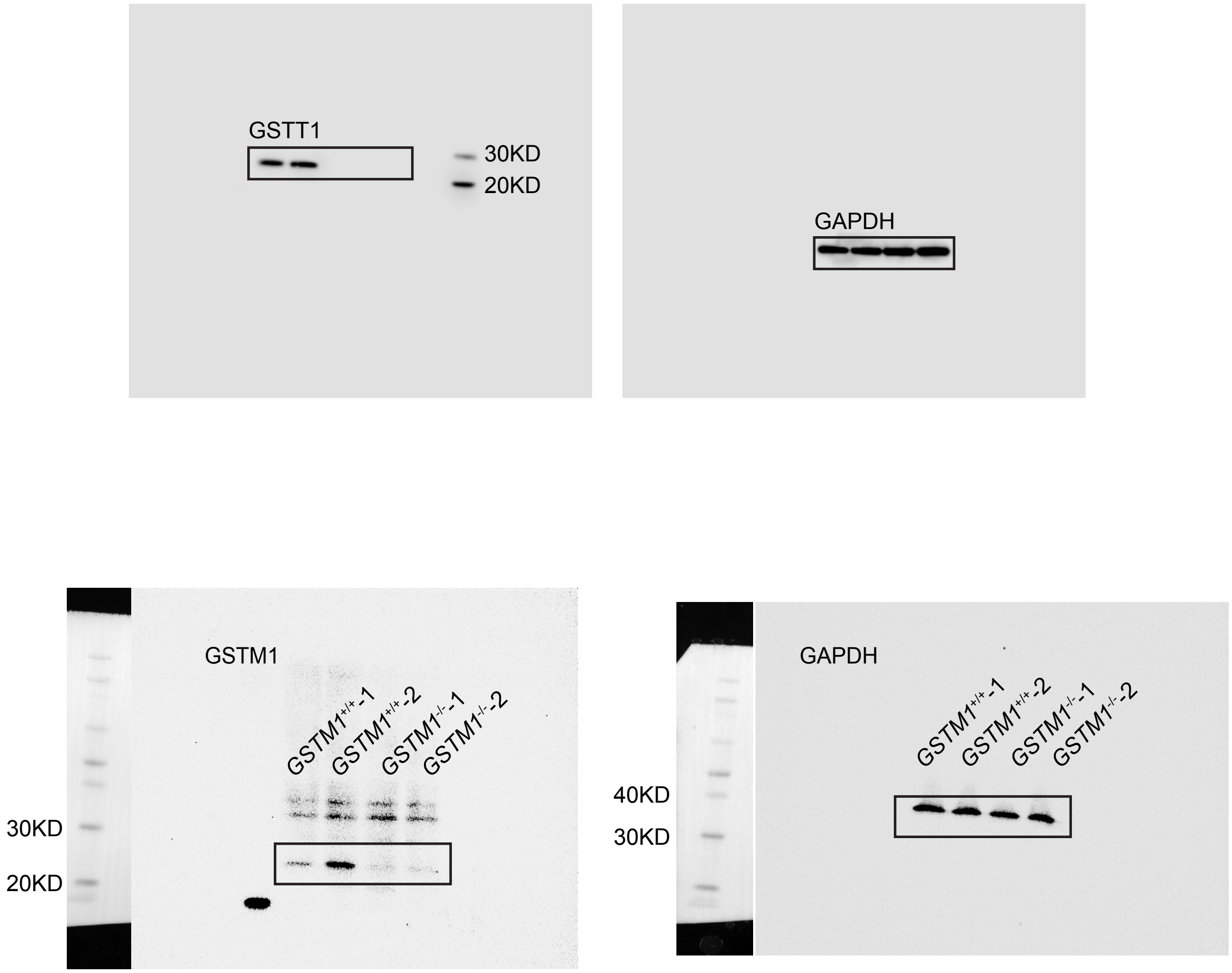
GSTT1
30KD
20KD
GAPDH
GSTM1
GSTM1+/+-1
GSTM1+/+-2
GSTM1-/--1
GSTM1-/--2
30KD
20KD
GAPDH
GSTM1+/+-1
GSTM1+/+-2
GSTM1-/--1
GSTM1-/--2
40KD
30KD

Fig. 3i

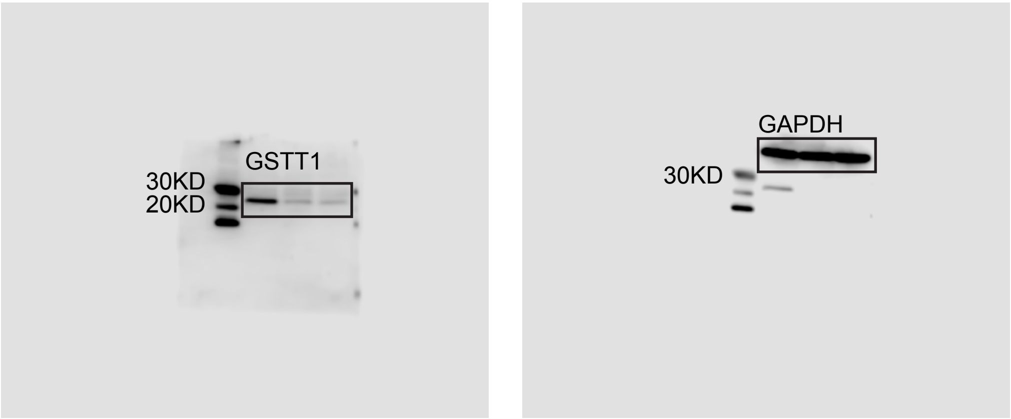
GSTT1
30KD
20KD
GAPDH
30KD

Supplementary Figure 7

Fig. 4f

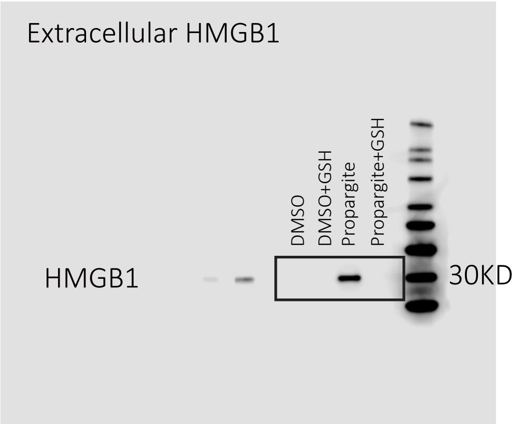

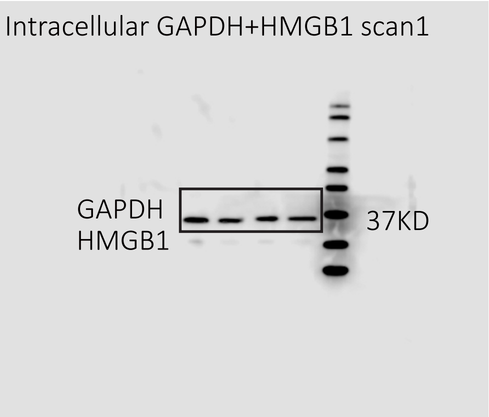

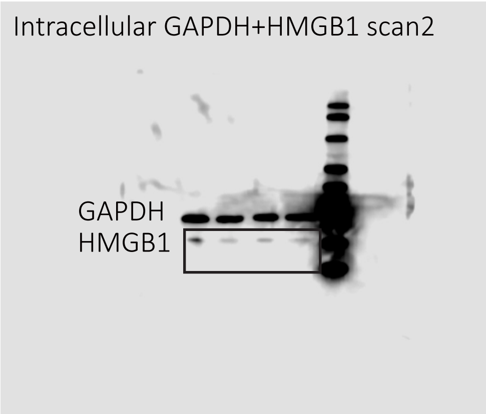

Supplementary Fig. 2f

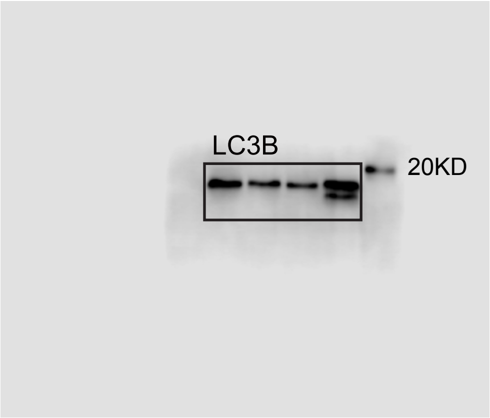

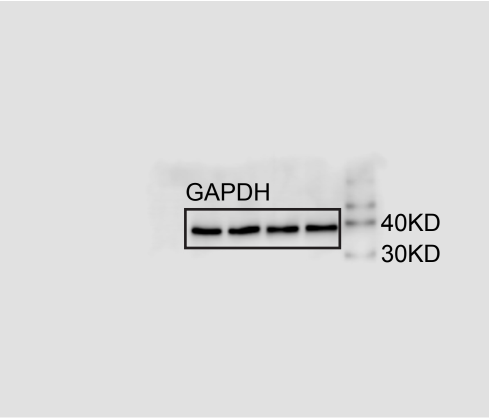

Supplementary Figure 7. Uncropped blot images for Figure 2c, 2d, 2e, 2m, 3d, 3i, 4f and Supplementary Fig. 2f

Supplementary Table 1. qRT-PCR primers.

Gene	Forward (5' to 3')	Reverse (5' to 3')
DDIT3	GGAAACAGAGTGGTCATTCCC	CTGCTTGAGCCGTTCATTCTC
GADD45A	CAATTAGTGTCGTGCGGCC	TTCTGCACTCACTCACAGGC

Supplementary Table 2. sgRNA sequences for gene targeting.

Gene	CRISPR	sgRNA sequence (5' of PAM)	Targeted cells
GSTM1	sg*GSTM1*	ATGGACAACCATATGCAGCT	hESCs
GSTT1	sg*GSTT1*-1	TGGGTCGGCCTTCGAAGACT	hESCs/ EndoC-βH1 cells
	sg*GSTT1*-2	CTGGCCATGATCCGGTGAGC	EndoC-βH1 cells

Supplementary Table 3. PCR and sequencing primers (Forward) used for genotyping the knockout hESC lines.

Gene	Forward (5' to 3')	Reverse (5' to 3')
GSTM1	CAACGCCATCTTGTGCTACA	GTGTCCTAAGGTTGGTGGGA
GSTT1	TGGTCCCCAAATCAGATGCT	AAAGGACACAAGGCCTCAGT

Supplementary Table 4. Efficiency for the creation of biallelic knockout hESC lines.

Gene	Sequenced sub-clone	Monoallelic knockout	Biallelic knockout	Biallelic knockout Efficiency
GSTM1	22	2	15	68%
GSTT1	14	0	10	71%

Supplementary Table 5. PCR primers for genomic DNA amplification.

	PCR primers (5' to 3')
HBB-F	GAAGAGCCAAGGACAGGTAC
HBB-R	CAACTTCATCCACGTTCACC
GSTM1-F	GAACTCCCTGAAAAGCTAAAGC
GSTM1-R	CTTGGGCTCAAATATACGGTGG
GSTT1-F	TTCCTTACTGGTCCTCACATCTC
GSTT1-R	TCACCGGATCATGGCCAGCA

Supplementary Table 1. qRT-PCR primers.

Gene	Forward (5' to 3')	Reverse (5' to 3')
DDIT3	GGAAACAGAGTGGTCATTCCC	CTGCTTGAGCCGTTCATTCTC
GADD45A	CAATTAGTGTCGTGCGGCC	TTCTGCACTCACTCACAGGC

Supplementary Table 2. sgRNA sequences for gene targeting.

Gene	CRISPR	sgRNA sequence (5' of PAM)	Targeted cells
GSTM1	sg*GSTM1*	ATGGACAACCATATGCAGCT	hESCs
GSTT1	sg*GSTT1*-1	TGGGTCGGCCTTCGAAGACT	hESCs/ EndoC-βH1 cells
	sg*GSTT1*-2	CTGGCCATGATCCGGTGAGC	EndoC-βH1 cells

Supplementary Table 3. PCR and sequencing primers (Forward) used for genotyping the knockout hESC lines.

Gene	Forward (5' to 3')	Reverse (5' to 3')
GSTM1	CAACGCCATCTTGTGCTACA	GTGTCCTAAGGTTGGTGGGA
GSTT1	TGGTCCCCAAATCAGATGCT	AAAGGACACAAGGCCTCAGT

Supplementary Table 4. Efficiency for the creation of biallelic knockout hESC lines.

Gene	Sequenced sub-clone	Monoallelic knockout	Biallelic knockout	Biallelic knockout Efficiency
GSTM1	22	2	15	68%
GSTT1	14	0	10	71%

Supplementary Table 5. PCR primers for genomic DNA amplification.

	PCR primers (5' to 3')
HBB-F	GAAGAGCCAAGGACAGGTAC
HBB-R	CAACTTCATCCACGTTCACC
GSTM1-F	GAACTCCCTGAAAAGCTAAAGC
GSTM1-R	CTTGGGCTCAAATATACGGTGG
GSTT1-F	TTCCTTACTGGTCCTCACATCTC
GSTT1-R	TCACCGGATCATGGCCAGCA
